# Anticoagulation for the Pregnant Patient with a Mechanical Heart Valve, No Perfect Therapy: Review of Guidelines for Anticoagulation in the Pregnant Patient

**DOI:** 10.1155/2017/3090273

**Published:** 2017-11-22

**Authors:** Aaron Richardson, Stuart Shah, Ciel Harris, Garry McCulloch, Patrick Antoun

**Affiliations:** ^1^Department of Medicine, University of Florida College of Medicine Jacksonville, Jacksonville, FL, USA; ^2^Department of Cardiology, University of Florida College of Medicine Jacksonville, Jacksonville, FL, USA

## Abstract

Heart valve replacement with a mechanical valve requires lifelong anticoagulation. Guidelines currently recommend using a vitamin K antagonist (VKA) such as warfarin. Given the teratogenic effects of VKAs, it is often favorable to switch to heparin-derived therapies in pregnant patients since they do not cross the placenta. However, these therapies are known to be less effective anticoagulants subjecting the pregnant patient to a higher chance of a thrombotic event. Guidelines currently recommend pregnant women requiring more than 5 mg a day of warfarin be switched to alternative therapy during the first trimester. This case report highlights a patient who was switched to alternative therapy during her first pregnancy and suffered a devastating cerebrovascular accident (CVA). Further complicating her situation was during a subsequent pregnancy; this patient continued warfarin use during the first trimester and experienced multiple transient ischemic attacks (TIAs). This case highlights the increased risk of thrombotic events in pregnant patients with mechanical valves. It also highlights the difficulty of providing appropriate anticoagulation for the pregnant patient who has experienced thrombotic events on multiple anticoagulants.

## 1. Introduction

Heart valve replacement with a prosthetic mechanical valve requires lifelong anticoagulation [1]. The annual risk of a thrombotic event in patients not taking anticoagulation is approximately 4% while the risk in those on appropriate anticoagulation is 1% [2]. Current guidelines recommend use of a vitamin K antagonist (VKA) with routine monitoring of prothrombin time (PT) and international normalized ratio (INR) to ensure therapeutic range for anticoagulation therapy. However, the teratogenic effects of VKAs make these medications unfavorable to use in pregnant women. During pregnancy, patients are often switched to alternative anticoagulation therapies which include low-molecular-weight heparin (LMWH) or unfractionated heparin (UFH), to avoid teratogenic effects from VKAs. However, heparin-derived anticoagulants have been proven to be not as effective as VKAs for anticoagulation with mechanical heart valves leaving the pregnant patient more vulnerable to a thrombotic event. Women and their physicians are left with the difficult decision of weighing the risk and benefits of anticoagulation during pregnancy. We present the case of a patient who was switched from warfarin to Lovenox during her first pregnancy and experienced a devastating bilateral middle cerebral artery (MCA) stroke which ultimately ended in the loss of her unborn child. The patient became pregnant a second time but continued warfarin through the first trimester. She had multiple transient ischemic attacks (TIA) while on therapeutic warfarin therapy. Her case is unusual in that she experienced two thrombotic events on two 2 different anticoagulants. Although no perfect therapy has been identified for the pregnant patient with a mechanical valve, guidelines and recommendations for providers are discussed.

## 2. Case Presentation

A 25-year-old African American married female with a past medical history of a previous pregnancy that resulted in a missed abortion, depression, bilateral MCA stroke, sickle cell trait, and mitral valve congenital defect replaced with St. Jude mechanical valve in 2001 presented at 11 weeks pregnant for management of high-risk pregnancy. The patient was diagnosed at birth with a mitral valve defect which was surgically repaired at age 2. However, at the age of 12, her native mitral valve was replaced with a St. Jude Master Series 27 mm mitral valve prosthesis. After replacement with mechanical mitral valve, she was prescribed oral warfarin therapy for anticoagulation. Her anticoagulation was managed by her cardiologist at an outside facility with a target INR of 2.5 to 3.5. The patient admitted to being compliant with her warfarin therapy. The patient only noted one prior ischemic event, a TIA at 12 years old, which was attributed to a missed medication dose. She said her mother forgot to give her the medication prior to the TIA. Her INRs were checked weekly although the patient did not have any lab work to present at the time of presentation. Approximately seven months prior to presentation, the patient became pregnant for her first time. She was switched from warfarin to Lovenox therapy to avoid teratogenic effects of warfarin to the fetus. Lovenox dosage was 60 mg (1 mg per kg dosing) administered twice daily, with antifactor Xa monitored by her cardiologist. The patient admitted to strict compliance to this anticoagulation regimen without any missed dosages. During her 8th week of pregnancy, the patient presented to the emergency department with a sudden onset of difficulty speaking and bilateral facial/upper extremity weakness. She was diagnosed with bilateral MCA infarcts and not deemed a candidate for intervention due to her pregnancy. During her admission for the stroke, the patient suffered a missed abortion. She was eventually discharged to a rehabilitation center for poststroke care and regained full motor and speech function. Approximately 3 months after her initial stroke, the patient became pregnant again. With this subsequent pregnancy, she was advised by her cardiologist to switch from warfarin therapy to Lovenox as she had done in the previous pregnancy. However, the patient refused given concern over another stroke even though she was aware of teratogenic risks to the unborn child. Although she was on a therapeutic dose of warfarin with regular INR checks, the patient had noted intermittent tingling around her mouth and numbness in her fingers during her first trimester, findings consistent with a TIA.

She was admitted to the hospital under the OBGYN service with consultation to cardiology for recommendations on anticoagulation. It was noted that she was on a daily dose of 10 mg warfarin, and her INR was therapeutic at 3.0 at the time of hospital admission. In anticipation of possible procedures, her warfarin was discontinued, and the patient was placed on a heparin infusion with goal activated partial thromboplastin time (aPTT) two times greater than the control. Electrocardiogram showed normal sinus rhythm. Given concern for valve thrombosis, transthoracic echo was ordered for further evaluation of mechanical valve. The transthoracic echo showed decreased posterior leaflet motion of the mechanical valve. As a result, transesophageal echo was performed which revealed normal valve motion and no evidence of thrombus. Normal atria and ventricle sizes were noted with left ventricular ejection fraction at 65–70%. Hematology was consulted for further workup for thrombophilia. Workup was unremarkable except she was noted to be heterozygous for MTHFR C677T gene. Homocysteine was within normal limits. Upon discharge, the patient was again advised on the need to switch to Lovenox therapy for anticoagulation; however, the patient refused and requested to be placed back on warfarin. Warfarin was restarted at 10 mg per day, and heparin drip was continued until therapeutic INR could be reached. The patient was discharged with follow-up with outside providers for INR checks and continued prenatal care. Since the patient did not follow up at our facility, it is unknown if she was able to deliver to term without any ischemic events or any effects to the fetus.

## 3. Discussion

Management of anticoagulation in pregnant women with mechanical heart valves is a difficult challenge for patients and physicians. Finding a delicate balance between adequate protection from a thrombotic event without causing harm to the unborn child is not easy. There are currently no clinical controlled trials to guide anticoagulation therapy for pregnant women; thus, no “optimal” therapy exists [3]. Current guidelines are based mostly on small retrospective series. Use of VKAs is the standard of treatment for prevention of valve thrombosis and embolic events in the general population of people who have received a mechanical heart valve [4, 5]. However, VKAs are capable of crossing the placental barrier and have teratogenic effects which include congenital abnormalities such as midfacial hypoplasia and stippling of epiphyses, along with central nervous system abnormalities such as hydrocephalus and optic atrophy. Stillbirth and miscarriage can also occur [6]. Although warfarin can pose a threat to the fetus at any point in the pregnancy, the fetus is most vulnerable to teratogenic effects during the first trimester. Previous case series have proven the embryopathy rate to be as high as 5 to 7% to those exposed to warfarin in the first trimester [7]. Some literature does suggest that these effects are dose dependent and that daily doses less than 5 mg/day appear to significantly reduce the risks of fetal toxicity [8, 9]. Vitale et al. demonstrated that 88% of women with a warfarin dose > 5 mg/day had fetal complications with 9% incident of warfarin embryopathy. In comparison, women with daily dose < 5 mg/day had a 15% chance of fetal complications, and none had warfarin embryopathy [8].

More recent data provide conflicting evidence of whether a safe dose of warfarin exists. McLintock et al. report several recent studies which suggest a dose relationship may exist for warfarin embryopathy, but no clear evidence could be found that warfarin embryopathy was dose related. Although a small number of cases were reported, there were five cases of warfarin embryopathy in women who were taking 5 mg or less of warfarin and seven cases in women who were taking more than 5 mg daily [10–14]. van Hagen et al. also reported no significant difference in fetal loss or miscarriage between high-dose warfarin (greater than 5 mg per day) and low-dose warfarin (5 mg per day or less) [15].

Although the greatest risk for teratogenic effects is in the first trimester, warfarin fetopathy is reported throughout all 3 trimesters in patients taking warfarin. Vitale et al. report miscarriage (loss less than 20 weeks gestation) and stillbirth (loss greater than 20 weeks gestation) rates to be as high as 63.6% and 15%, respectively, in women taking daily warfarin doses greater than 5 mg. This is compared to a miscarriage and a stillbirth rate of 5.2% and 0%, respectively, in women taking a daily warfarin dose of 5 mg or less [8]. Soma-Pillay et al. also reported increasing rates of miscarriage and stillbirth in women with increased dosages of warfarin [11].

The literature recommends switching to a LMWH for anticoagulation from the beginning of pregnancy through the first 12 weeks (end of first trimester). LMWH is preferred during the first trimester as it does not cross the placenta. However, LMWH is not as effective as VKAs in prevention of a thrombotic event; thus, a higher risk of maternal complications exists. UFH is another option but has also been shown to be inferior to VKAs for anticoagulation and requires inpatient admission. Ginsberg et al. report the incident of thrombotic events in pregnant women with mechanical heart valves to be 3.9% in pregnancies of women taking warfarin throughout the pregnancy, 9.2% in pregnancies of women who received UFH in the first trimester followed by warfarin, and 33% in pregnancies treated with UFH throughout the pregnancy [16]. Meschengieser et al. conducted a prospective cohort study which included 92 pregnant patients with mechanical heart valves. This study compared oral anticoagulation throughout with first trimester to subcutaneous heparin 12,500 units every 12 hours. It revealed a thrombotic rate of 4.92% with heparin and a 0.33% rate with warfarin [17]. Smaller reviews have examined the rate of thrombotic events in pregnant women receiving LMWH treatment. McLintock et al. report the rate of thrombosis in women receiving LMWH to be 6.9%; however, this number was not based on studies in which 5 or more pregnancies were included [18]. Larger studies are required to gain a better understanding of risk of thrombotic events in pregnant patients.

For patients in which LMWH is used, monitoring of antifactor Xa levels is recommended to ensure therapeutic range of anticoagulation. This is reflected in the current American College of Cardiology/American Heart Association (ACC/AHA) guidelines. However, manufacturers recommend that both peak and trough levels should be measured. Per review of the literature, it is recommended that peak anti-Xa levels be measured 4 to 6 hours after administration with target anti-Xa level of 1.0 IU/ml to 1.2 IU/ml. Trough anti-Xa levels of 0.6–0.7 IU/ml are recommended. Peak anti-Xa levels should not exceed 1.5 IU/ml [19]. Peak and trough levels are both noted to be necessary to ensure optimum therapy. Multiple authors have examined anti-Xa peak and trough levels and found most patients to have subtherapeutic trough levels. Elkayam and Goland examined anti-Xa trough and peak levels in 30 pregnant patients who had their anticoagulation switched to LMWH during their entire pregnancy. Eight patients were noted to be on anticoagulation for mechanical heart valves. A total of 187 paired peak and trough levels were obtained. The recommended peak anti-Xa level (0.7–1.2 U/ml) occurred in only 66% of the measurements, and 80% of trough levels were found to be subtherapeutic (<0.6 U/ml) [15]. No complications were noted in patients with mechanical valves. Quinn et al. examined LMWH dosing in 11 women with 12 pregnancies who had mechanical heart valves. Upon initiation of therapy, the women were started on twice daily dosing of LMWH at a dose of 1 mg/kg with subsequent monitoring of anti-Xa levels. They found a mean increase in dose of LMWH of 54.4% from base dosage was needed to achieve therapeutic anti-Xa levels [20]. Other authors have also demonstrated large numbers of subtherapeutic anti-Xa levels in pregnant patients using LMWH for anticoagulation [21, 22]. Subtherapeutic anti-Xa levels are known to place patients at an increased risk for thrombotic events [2]. Although these data support the need for routine monitoring of peak and trough anti-Xa levels, more prospective studies are needed. The ACC/AHA guidelines do not provide any guidance on measuring peak and trough anti-Xa levels at this time.

Low-dose aspirin (75 mg to 100 mg) is recommended as an addition to VKA therapy during second and third trimesters for patients at a higher risk for embolic events and is included in the ACC/AHA guidelines. van Hagen et al. examined the use of low-dose aspirin therapy in the second and third trimesters. Out of 212 pregnant patients with mechanical heart valves, only 13 were given low-dose aspirin as add-on therapy during pregnancy. Out of these patients, none experienced a thrombotic event in the second or third trimester as opposed to 5 out of the remaining 199 who did not receive aspirin but had an event. Hemorrhagic events were noted in 8 of the 13 patients who received aspirin therapy (61.5%), and 41 of the 199 patients (20.6%) experienced hemorrhagic events in the no aspirin therapy group [15]. It was unclear how serious these hemorrhagic events were. It would appear aspirin does provide additional protection against ischemic events but does come with an increased risk of hemorrhage. Other studies with small number of patients have also shown a decrease in thrombotic events with adding aspirin; however, an increased risk of bleeding was noted in these patients as well [12, 18]. Given the limited number of patients in which the addition of aspirin therapy has been studied, it is not possible at this time to conclude whether the benefits of aspirin therapy outweigh the risk of hemorrhage.

The current guidelines released in 2014 from the ACC/AHA do have recommendations for anticoagulation in patients with mechanical heart valves and specifically address management in pregnant patients. Class I recommendations are as follows. For all patients taking a VKA, such as warfarin, a therapeutic INR of 2-3 (INR of 2.5–3.5 for mitral valves) is recommended. In pregnant patients, warfarin may be used to achieve therapeutic INR in second and third trimesters. It is also recommended to discontinue warfarin and initiate IV UFH with aPTT greater than two times the control before planned vaginal delivery. Low-dose aspirin (75 mg to 100 mg) is also recommended during second and third trimesters [5, 23]. No Class I recommendations are available for anticoagulation during the first trimester. (Please see Table 1 for full ACC/AHA guidelines.)

Planned delivery is recommended in all patients unless obstetric indications call for caesarean section. It is recommended that all women on VKA switch to LMWH or UFH at week 34–36 with induction or caesarean at around 38 weeks. UFH should be started approximately 24 hours prior to labor induction or caesarean with target aPTT at 2-3 times above baseline. IV UFH should be stopped once in labor or 6 hours prior to anesthesia administration confirming aPTT back to baseline. UFH should be restarted 4 to 6 hours post vaginal delivery or 6–12 hours post caesarean section with IV UFH at 500 IU/hour and increase over 24–48 hours to target aPTT. Warfarin should be restarted on day 1 postpartum (uncomplicated vaginal delivery) or on day 2-3 if caesarian or bleeding complications occur [18].

Currently, there is no indication for the use of direct acting oral anticoagulants (DOACs) for anticoagulation in patients who have received a mechanical heart valve. Previous studies have shown that there is an increased risk of thrombotic events and bleeding when using a DOAC [24, 25]. Currently DOACs are not recommended for use in pregnant patients as they are able to cross into the placenta. Further study is needed to understand the effects of these medications [26].

As for pregnant patients who suffer a thrombotic event while on appropriate anticoagulation therapy, there are no clear options for treatment. Surgical interventions are not recommended. However, an option for treating thrombosis during pregnancy is use of thrombolytic medications such as streptokinase or urokinase for up to 72 hours with no negative effects on fetus; however, the data are very limited [27]. There were no guidelines available to guide physicians in managing patients who have failed multiple anticoagulation regimens and suffered an ischemic event.

## 4. Conclusions

Anticoagulation is a necessity for the pregnant patient with a mechanical heart valve. However, these women along with their physician are left with weighing risks to the mother and unborn child. Before an anticoagulation regimen is decided upon, risks should be explained to the expectant patient and family. Based on the literature and current guidelines, continuation of VKA therapy may be appropriate through the pregnancy if dosage is less than 5 mg a day. Since dosing is based on the amount needed to obtain a therapeutic INR, no guarantee can be made that a higher dose could be required during pregnancy. The decisions become even more difficult with a daily dose greater than 5 mg a day. The patient presented in this case is unusual as she experienced thrombotic events on 2 different anticoagulation therapies, one which included a VKA with a therapeutic INR. Although the patient suffered TIA symptoms during the second pregnancy, she was lost to follow-up; thus, it still remains unclear if she made it to term, suffered an ischemic event, or had other complications. There is also no clinical indication to perform a hypercoagulable workup in this patient population since there are no data to support a higher risk of thrombosis in patients with a mechanical heart valve who have a thrombophilia versus those who do not. Guidelines are unclear how to approach anticoagulation in pregnant patients who have experienced a thrombotic event while on guideline-directed therapy. Although aspirin could have been added to this patient's therapy, there are no significant data pointing to an improved outcome as a much higher risk of hemorrhage has been noted. All women of childbearing age requiring anticoagulation for a mechanical valve should be counseled on the risk of a thrombotic event which could result in serious illness or death to her or the unborn child. As for patients who have suffered an ischemic event, even on a VKA, the answers become even more difficult.

## Figures and Tables

**Table 1:** ACC/AHA anticoagulation guidelines for pregnant patients with mechanical heart valves.

Class I	Class IIa	Class IIb	Class III
A therapeutic INR of 2-3 (INR of 2.5–3.5 for mitral valves) for all patients prescribed a VKA	Continue VKA during first trimester in pregnant patients if dose to achieve therapeutic INR is 5 mg per day or less with full discloser of risks	If daily warfarin dose less than or equal to 5 mg a day to achieve therapeutic INR, adjusted LMWH at least two times a day with target anti-Xa level of 0.8 IU/ml to 1.2 IU/ml 4 to 6 hours after administration may be used during first trimester of pregnancy	LMWH should not be administered unless anti-Xa levels are monitored 4 to 6 hours after administration.

In pregnant patients, warfarin may be used to achieve therapeutic INR in second and third trimesters	LMWH at least two times a day with target anti-Xa level of 0.8 IU/ml to 1.2 IU/ml 4 to 6 hours after administration during first trimester if daily warfarin dose greater than 5 mg per day for therapeutic INR	Dose adjusted continuous intravenous UFH with aPTT at least 2 times greater than control for pregnant patients with warfarin dose less than or equal to 5 mg a day for therapeutic INR during the first trimester	—

Discontinue VKA and initiate IV UFH with activated partial thromboplastin time (aPTT) greater than two times the control before planned vaginal delivery	Adjusted continuous intravenous UFH with aPTT at least 2 times greater than control for pregnant patient if daily warfarin dose greater than 5 mg a day for therapeutic INR	—	—

Add low-dose aspirin (75 mg to 100 mg) during second and third trimesters	—	—	—

Class I: treatment should be performed or administered. Class IIa: it is reasonable to perform procedure or administer treatment. Additional studies with focused objectives needed. Class IIb: procedure or treatment may be considered. Additional studies with broad objectives needed. Class III: procedure or treatment should not be performed or administered since it is not helpful and may be harmful.

## References

[1] McGoon M. D., Fuster V., McGoon D. C., Pumphrey C. W., Pluth J. R., Elveback L. R. (1984). Aortic and mitral valve incompetence: long-term follow-up (10 to 19 years) of patients treated with the Starr-Edwards prosthesis. *Journal of the American College of Cardiology*.

[2] McLintock C. (2011). Anticoagulant therapy in pregnant women with mechanical prosthetic heart valves: no easy option. *Thrombosis Research*.

[3] Aguilera C., Agustí A. (2003). Anticoagulation of pregnant women with mechanical heart valves. *Medicina Clinica*.

[4] Hirsh J., Fuster V., Ansell J., Halperin J. L. (2003). American Heart Association/American College of Cardiology foundation guide to warfarin therapy. *Circulation*.

[5] Nishimura R. A., Otto C. M., Bonow R. O. (2017). 2017 AHA/ACC focused update of the 2014 AHA/ACC guideline for the management of patients with valvular heart disease. *Journal of the American College of Cardiology*.

[6] Castellano J. M., Narayan R. L., Vaishnava P., Fuster V. (2012). Anticoagulation during pregnancy in patients with a prosthetic heart valve. *Nature Reviews Cardiology*.

[7] Economy K. E., Valente A. M. (2015). Mechanical heart valves in pregnancy: a sticky business. *Circulation*.

[8] Vitale N., De Feo M., De Santo L. S., Pollice A., Tedesco N., Cotrufo M. (1999). Dose-dependent fetal complications of warfarin in pregnant women with mechanical heart valves. *Journal of the American College of Cardiology*.

[9] Xu Z., Fan J., Luo X. (2016). Anticoagulation regimens during pregnancy in patients with mechanical heart valves: a systematic review and meta-analysis. *Canadian Journal of Cardiology*.

[10] McLintock C. (2014). Thromboembolism in pregnancy: challenges and controversies in the prevention of pregnancy-associated venous thromboembolism and management of anticoagulation in women with mechanical prosthetic heart valves. *Best Practice & Research Clinical Obstetrics & Gynaecology*.

[11] Soma-Pillay P., Nene Z., Mathivha T. M., Macdonald A. P. (2011). The effect of warfarin dosage on maternal and fetal outcomes in pregnant women with prosthetic heart valves. *Obstetric Medicine*.

[12] Yinon Y., Siu S. C., Warshafsky C. (2009). Use of low molecular weight heparin in pregnant women with mechanical heart valves. *American Journal of Cardiology*.

[13] Mazibuko B., Ramnarain H., Moodley J. (2012). An audit of pregnant women with prosthetic heart valves at a tertiary hospital in South Africa : a five-year experience. *Cardiovascular Journal of Africa*.

[14] Cotrufo M., De Feo M., De Santo L. S. (2002). Risk of warfarin during pregnancy with mechanical valve prostheses. *Obstetrics & Gynecology*.

[15] van Hagen I. M., Roos-Hesselink J. W., Ruys T. P.E. (2015). Pregnancy in women with a mechanical heart valve: data of the European Society of Cardiology Registry of Pregnancy and Cardiac disease (ROPAC). *Circulation*.

[16] Ginsberg J. S., Chan W. S., Bates S. M., Kaatz S. (2003). Anticoagulation of pregnant women with mechanical heart valves. *Archives of Internal Medicine*.

[17] Meschengieser S. S., Fondevila C. G., Santarelli M. T., Lazzari M. A. (1999). Anticoagulation in pregnant women with mechanical heart valve prostheses. *Heart*.

[18] McLintock C., McCowan L. M. E., North R. A. (2009). Maternal complications and pregnancy outcome in women with mechanical prosthetic heart valves treated with enoxaparin. *BJOG: An International Journal of Obstetrics & Gynaecology*.

[19] Elkayam U., Goland S. (2012). The search for a safe and effective anticoagulation regimen in pregnant women with mechanical prosthetic heart valves. *Journal of the American College of Cardiology*.

[20] Quinn J., Von Klemperer K., Brooks R., Peebles D., Walker F., Cohen H. (2009). Use of high intensity adjusted dose low molecular weight heparin in women with mechanical heart valves during pregnancy : a single-center experience. *Haematologica*.

[21] Barbour L. A., Oja J. L., Schultz L. K. (2004). A prospective trial that demonstrates that dalteparin requirements increase in pregnancy to maintain therapeutic levels of anticoagulation. *American Journal of Obstetrics and Gynecology*.

[22] Friedrich E., Hameed A. B. (2009). Fluctuations in anti-factor Xa levels with therapeutic enoxaparin anticoagulation in pregnancy. *Journal of Perinatology*.

[23] Nishimura R. A., Otto C. M., Bonow R. O. (2014). 2014 AHA/ACC guideline for the management of patients with valvular heart disease a report of the American College of Cardiology/American Heart Association task force on practice guidelines. *Circulation*.

[24] Shazly A., Afifi A. (2014). RE-ALIGN: first trial of novel oral anticoagulant in patients with mechanical heart valves–the search continues. *Global Cardiology Science and Practice*.

[25] Granger C. B., Eikelboom J. W., Connolly S. J. (2013). Dabigatran versus warfarin in patients with mechanical heart valves. *New England Journal of Medicine*.

[26] Weitz J. I. (2014). Expanding use of new oral anticoagulants. *F1000Prime Reports*.

[27] Pieper P. G., Balci A., Van Dijk A. P. (2008). Pregnancy in women with prosthetic heart valves. *Netherlands Heart Journal*.

